# Clomiphene citrate throughout the duration of ovarian stimulation in patients with diminished ovarian reserve: an approach to decrease costs, reduce injection burden, and prevent premature ovulation

**DOI:** 10.1007/s10815-025-03412-w

**Published:** 2025-02-01

**Authors:** Rachel S. Mandelbaum, Samuel Melville, Aaron Masjedi, Natasha Raj-Derouin, Intira Sriprasert, Molly M. Quinn, Richard J. Paulson, John G. Wilcox, Joie Z. Guner

**Affiliations:** 1 Division of Reproductive Endocrinology and Infertility, Department of Obstetrics and Gynecology, University of Southern California Keck School of Medicine, 2020 Zonal Avenue, IRD520, Los Angeles, CA 90033 USA; 2 HRC Fertility, an affiliate of Keck School of Medicine at USC, Pasadena, CA USA; 3 USC Keck School of Medicine, Los Angeles, CA USA; 4 Department of Obstetrics and Gynecology, Kaiser Permanente Los Angeles Medical Center, Los Angeles, CA USA

**Keywords:** Clomiphene citrate, Diminished ovarian reserve, In vitro fertilization, Premature ovulation

## Abstract

**Purpose:**

Clomiphene citrate (CC) is often utilized as an adjunct in in vitro fertilization (IVF) protocols during the first 5 days of stimulation for endogenous FSH release. However, due to its antiestrogenic mechanism of action, CC may also effectively prevent the LH surge, and hence premature ovulation, if continued until the day of trigger. The objective of this study was to evaluate a “long CC” protocol, in which CC is continued throughout the entire cycle in-lieu of GnRH antagonist, and to compare IVF outcomes with a standard 5-day CC + GnRH antagonist protocol in patients with diminished ovarian reserve (DOR) undergoing IVF with high-dose gonadotropins.

**Methods:**

This is a retrospective cohort study of all CC-based IVF cycles at a single institution between 9/2020 and 9/2022. Mild stimulation protocols were excluded. The long CC group received CC throughout the entire cycle without GnRH antagonist. The CC + GnRH antagonist group received CC for the first 5 days of stimulation followed by GnRH antagonist when the lead follicle reached 14 mm. The primary outcome was mature oocyte yield.

**Results:**

There were 361 cycles (77%) in the long CC group and 108 (23%) in the 5-day CC + GnRH antagonist group. Age and AMH levels were similar between the two groups. There was no significant difference in mature oocyte yield between the long CC and 5-day CC + GnRH antagonist groups (median 5 (IQR 5) vs. 4.5 (IQR 5), respectively, (*P* = 0.922)). MII oocytes/AFC did not differ (0.69 vs. 0.56, respectively, *P* = 0.16). Premature ovulation occurred in 0.3% of cycles in the long CC group vs. 3.0% of cycles in the 5-day CC + GnRH antagonist group (*P* = 0.019).

**Conclusions:**

In DOR patients undergoing IVF, a long CC protocol is an effective and patient-friendly approach associated with non-inferior oocyte yield.

## Introduction

Clomiphene citrate (CC) was one of the original agents used for controlled ovarian stimulation for in vitro fertilization (IVF) in the 1980s [1]. It is a competitive antagonist of the estrogen receptor in the hypothalamus and anterior pituitary, leading to an increase in gonadotropin release. Over 40 years of data have confirmed the safety and efficacy of CC, and it is now widely available and inexpensive for patients [1–5].

CC is still often used as an adjunct during ovarian stimulation in patients anticipated to have a poor response. In these protocols, CC is often administered during the first 5 days of ovarian stimulation, mimicking the regimen for ovulation induction or superovulation [6–9]. In this capacity, blockade of the negative feedback loop between estradiol and the anterior pituitary leads to increased endogenous release of both follicle-stimulating hormone (FSH) and luteinizing hormone (LH), adding to exogenous recombinant gonadotropin dosing. Gonadotropin-releasing hormone (GnRH) antagonists are typically added to prevent premature ovulation prior to oocyte retrieval.

In addition to the blockade of the negative feedback loop, CC may also sufficiently inhibit the positive feedback loop between estradiol and the anterior pituitary, which is necessary for the endogenous LH surge and ovulation. Historical pharmacodynamic studies do suggest CC’s ability to block the LH surge and prevent ovulation in the late follicular phase [2, 4, 5]. This suggests that there may be an added role for CC in ovarian stimulation protocols to prevent premature ovulation, potentially replacing the need for subcutaneous GnRH antagonist injections. This is particularly relevant for poor responders who may benefit from the additional gonadotropin stimulation, are at a heightened risk of premature ovulation, and often require multiple cycles with significant financial burden to achieve success.

The objective of this study is to compare a novel “long CC protocol,” in which CC is administered throughout the entire duration of ovarian stimulation in lieu of GnRH antagonist, with the traditional 5-day CC + GnRH antagonist protocol in poor responder patients undergoing stimulation with high dose recombinant follicle stimulating hormone (rFSH) who are anticipated to have a poor response. We hypothesize that this long CC protocol may yield a noninferior number of oocytes and effectively prevent premature ovulation, and provide a more patient-friendly alternative to GnRH antagonist cycles by decreasing both patient costs and injection burden.

## Methods

This is a retrospective cohort study of patients treated at the University of Southern California Fertility and Huntington Reproductive Center (HRC Fertility) in Pasadena, California, between September 2020 and September 2022. These two centers merged during the study period into a combined laboratory, and patients treated before and after the transition were included. Patients with anticipated poor response to ovarian stimulation undergoing controlled ovarian stimulation for IVF and intracytoplasmic sperm injection (ICSI) with CC-based protocols were included. Protocol selection was per provider preference based on patient anti-mullerian hormone (AMH) results, antral follicle count (AFC), and/or prior response to ovarian stimulation. Minimal stimulation protocols were excluded.

**Fig. 1:**
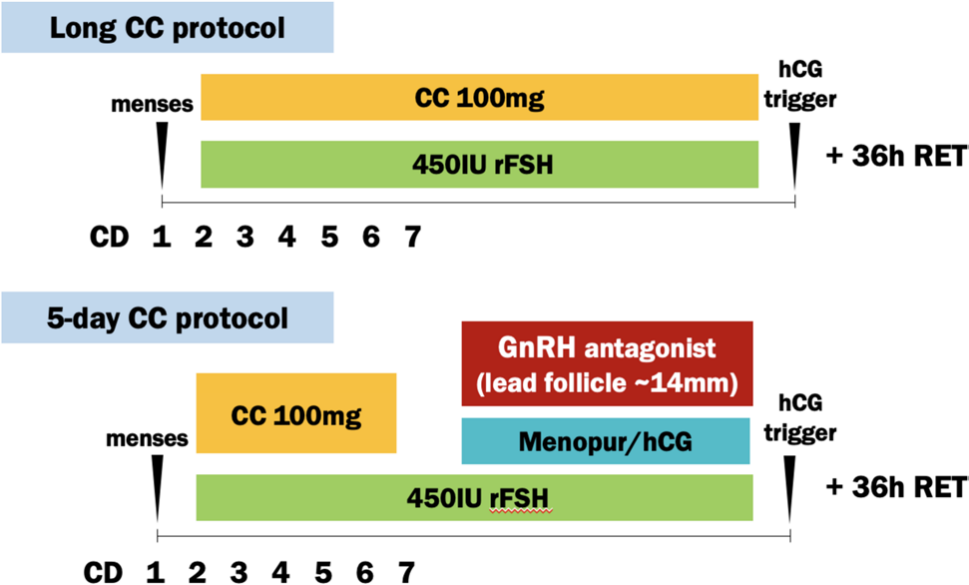
Schematic of CC-based ovarian stimulation protocols. Legend: Illustration of two CC-based protocols in the study. CD, cycle day; RET, oocyte retrieval

A schematic of the two protocols is shown in Fig. 1. Stimulation was started on cycle days 2 or 3. The long CC protocol participants received 100 mg of CC orally throughout the entire duration of stimulation as compared to only for the first 5 days of stimulation in the 5-day CC + GnRH antagonist group. Maximal rFSH (450 IU) dosing for the clinic was given in both groups, either with Follistim ® or Gonal-f ® nightly subcutaneous injections. Nightly GnRH antagonist (ganirelix or cetrotide) was added in the 5-day CC + GnRH antagonist group when the lead follicle reached approximately 14 mm. On this same day, patients in the 5-day CC + GnRH antagonist group additionally received medications containing LH activity, either in the form of Menopur ® (Ferring Pharmaceuticals) or low-dose human chorionic gonadotropin (hCG). Patients in the long CC group did not receive any exogenous medications with LH activity, as this was accomplished endogenously with pituitary LH release in response to CC.

Patients received regular monitoring ultrasounds every 2–3 days after the fifth day of stimulation to quantify follicular growth and serum estradiol levels. Either 5000 IU or 10,000 IU of hCG was given to trigger final oocyte maturation. Progesterone levels were obtained on the day of trigger injection. All patients had a 36-h trigger-to-retrieval window. The type of embryo culture/transfer and utilization of preimplantation genetic testing for aneuploidy (PGT-A) were per provider preference. Intracytoplasmic sperm injection was performed in all patients. All patients who received the long CC protocol underwent blastocyst culture with PGT-A. In contrast, only 22% of patients in the 5-day CC + GnRH antagonist group had blastocyst culture with PGT-A, and the majority (78%) had a fresh day 3 embryo transfer.

The primary outcome of the study was the number of mature, i.e., metaphase II oocytes retrieved. Given that number of oocytes retrieved depends greatly on an individual patient’s ovarian reserve and cannot necessarily be generalized from patient-to-patient, an additional metric correcting for the starting AFC was analyzed (number of mature oocytes per starting AFC). Secondary outcomes included cycle characteristics and outcomes, embryo development, premature ovulation, cycle cancellation, and live birth rate. Cycle characteristics and outcomes included length of stimulation, peak estradiol levels, number of follicles $\ge$ 14 mm on the day of trigger injection, progesterone level on the day of trigger injection, endometrial thickness on the day of trigger injection, and total number of oocytes retrieved. Embryo development included the fertilization rate, defined as the number of embryos with two pronuclei and two polar bodies out of the total number that underwent ICSI, and blastulation rate (for embryos that were cultured to blastocyst), defined as the number of mature blastocysts graded 3CC or better between days 5 and 7 of embryo culture per the number fertilized. Euploidy rate was also evaluated, defined as the number of euploid embryos divided by the total number of blastocysts biopsied per cycle.

Premature ovulation was defined as clinical evidence of ovulation by follicular collapse and progesterone rise. LH was not measured.

Categorical variables were analyzed with the chi-squared test. Normally distributed continuous variables were analyzed with Student’s *t*-test, while variables that were non-normally distributed were analyzed with the Mann–Whitney *U*-test. For the multivariable analysis, linear regression was used controlling for age, race, body mass index, AMH, AFC, and gravidity.

## Results

There were 361 (77%) patients who received the long CC protocol as compared to 108 (23%) who received the 5-day CC + GnRH antagonist protocol. Patient demographic variables are shown in Table 1. There was no difference in mean age or AMH; however, the mean AFC at the baseline ultrasound at the start of stimulation was slightly but statistically significantly lower in the long CC group as compared to the 5-day CC + GnRH antagonist group (8.1 vs. 9.2, respectively, *P* = 0.026) (Table 1).

**Table 1:** Patient demographics

	**Long CC protocol** ***n* = 361 (77%)**	**5-day CC** + GnRH antagonist **protocol** *n* = 108 (23%)	***P*-value**
Age (mean, SD)	39.4 (3.8)	40.2 (3.9)	0.061
Race/ethnicity (*n*, %)			** < 0.001**
White	108 (29.9)	72 (66.7)	
Black	13 (3.6)	4 (3.7)	
Asian	188 (52.1)	21 (19.4)	
Hispanic	40 (11.1)	6 (5.6)	
Other	12 (3.3)	5 (4.6)	
AMH (mean, SD)	1.1 (1.1)	1.1 (0.8)	0.745
Antral follicle count (AFC) at start of stimulation (mean, SD)	8.1 (3.1)	9.2 (4.9)	**0.026**
Body mass index (kg/m^2^) (mean, SD)	22.8 (3.2)	23.3 (3.1)	0.458
Gravidity (*n*, %)			0.104
0	155 (42.9)	36 (33.3)	
1	162 (44.8)	52 (48.1)	
2 +	44 (12.2)	20 (18.5)	
Parity (*n*, %)			**0.025**
0	194 (53.7)	42 (38.9)	
1	144 (39.9)	57 (52.8)	
2 +	23 (6.4)	9 (8.3)	

Legend: Demographic variables are presented as described. *P*-values obtained with chi-squared tests for categorical variables and Student’s *t*-test for continuous variables. Statistically significant *P-*values are presented in bold

**Fig. 2:**
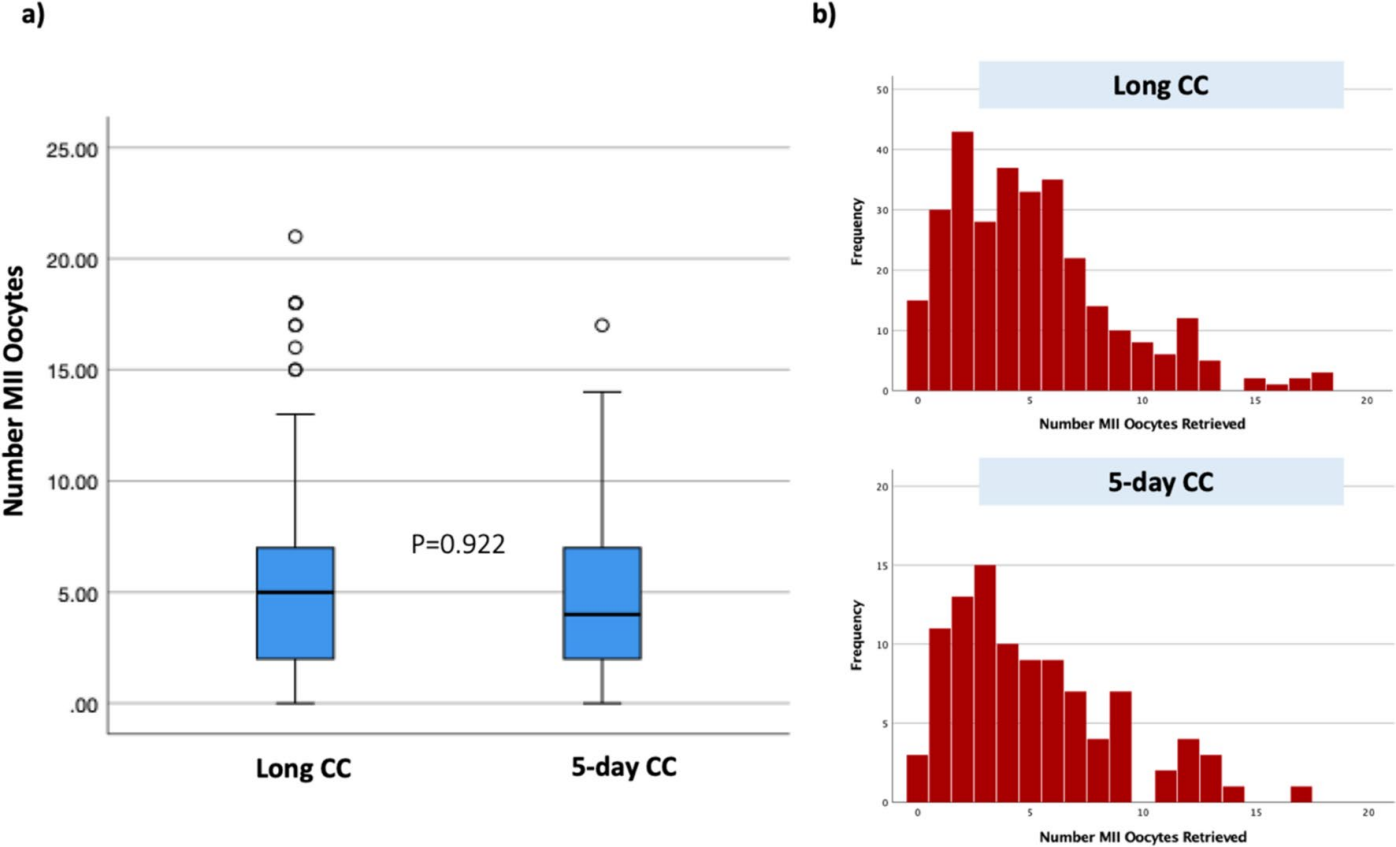
Comparison of mature oocyte yield between the long CC and 5-day CC + GnRH antagonist groups. Legend: **a** Comparison of median mature oocyte yield between the two groups. *P-value obtained with Mann Whitney *U*-test. **b** Histograms for number of mature oocytes retrieved in the two groups. Y-axis depicts frequency and x-axis depicts number of mature oocytes retrieved

**Fig. 3:**
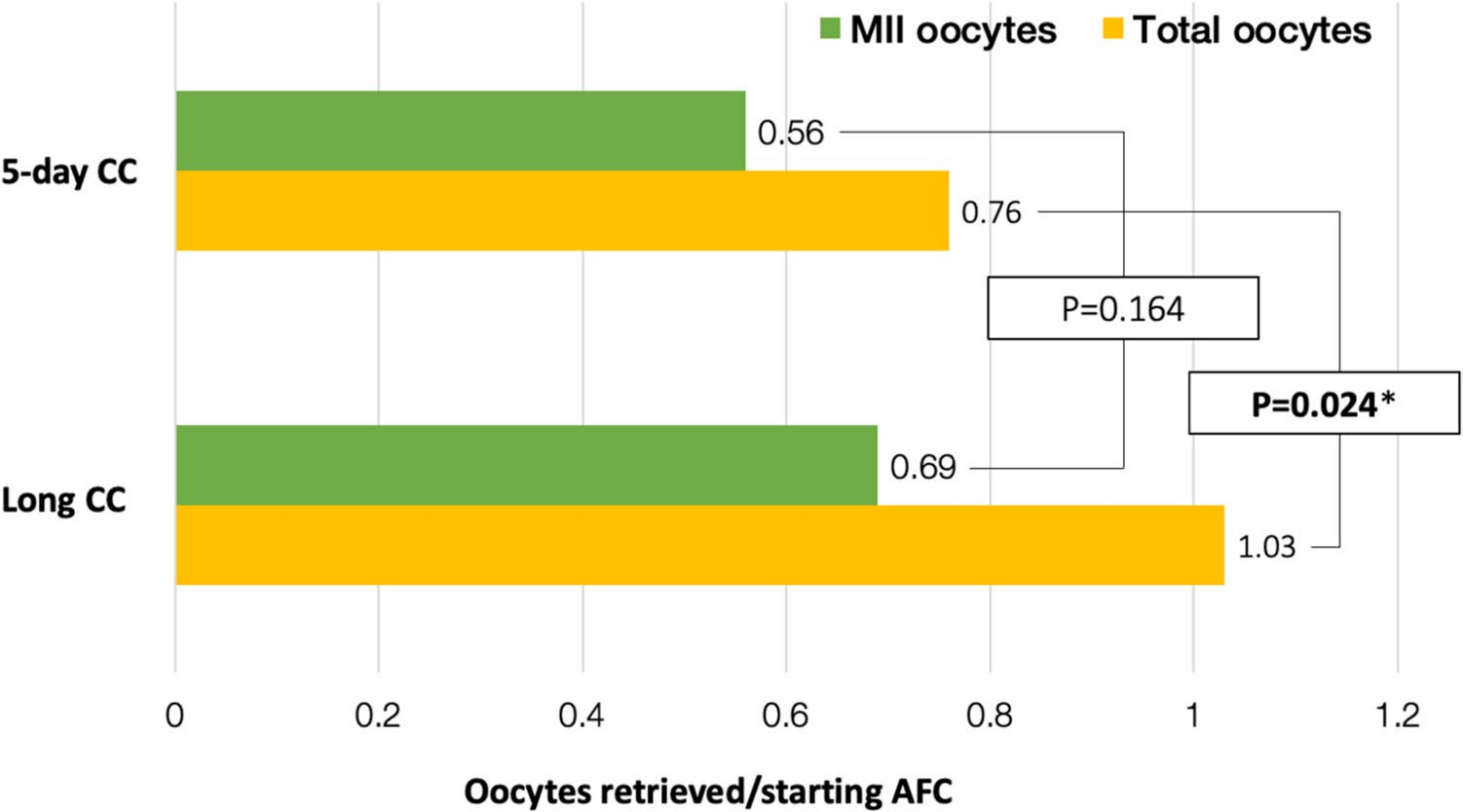
Oocyte yield by baseline antral follicle count. Legend: Number of mature oocytes divided by the baseline antral follicle count (AFC) at the start of the cycle. Green depicts mature oocytes and yellow depicts total number of oocytes including immature oocytes. *P*-vales obtained by Student’s *t*-test

Cycle characteristics are shown in Table 2. There was no difference in length of stimulation between the two groups. Hormonal profiles, including estrogen and progesterone levels, during stimulation, were significantly higher in the long CC group. Interestingly, there was no difference in endometrial thickness on the day of trigger between the two groups. While the long CC group had a statistically significantly greater mean number of follicles $\ge$ 14 mm on trigger day (*P* = 0.001, β coefficient 0.130) and retrieved a higher number of oocytes (*P* = 0.011, β coefficient 0.102), the number of mature oocytes was not different between the two groups (Fig. 2). Figure 3 illustrates oocyte yield outcomes by patient-specific starting AFC. While total number of oocytes retrieved also increased in the long CC group, this again did not translate to a greater number of mature oocytes retrieved per starting AFC. A post-hoc power calculation was performed using an alpha of 0.05 with the means, standard deviations, and number of subjects in each group for the primary outcome, number of mature oocytes divided by the starting AFC, which showed an 82.8% power to detect a difference.

**Table 2:** Cycle outcomes

	**Long CC protocol**	**5-day CC** + GnRH antagonist **protocol**	***P*-value**
Stimulation days (mean, SD)	10.0 (2.1)	10.4 (1.7)	0.060
Estradiol on trigger day (pg/mL) (mean, SD)	2,511.1 (1,478.8)	1,864.6 (1,073.6)	** < 0.001**
Progesterone on trigger day (ng/mL) (mean, SD)	1.2 (0.7)	0.8 (0.5)	** < 0.001**
Endometrial thickness on trigger day (mm) (mean, SD)	8.2 (2.1)	8.5 (2.1)	0.259
Number of follicles on US $\ge$ 14 mm on trigger day (median, interquartile range)	7.0 (5.0)	5.0 (4.0)	**0.001**
Total number of oocytes retrieved (median, interquartile range)	7.0 (5.0)	5.0 (4.0)	**0.011**
Number of mature oocytes retrieved (median, interquartile range)	5.0 (5.0)	4.5 (5.0)	0.934
Fertilization rate (%) (mean, SD)	72.9 (20)	70.3 (22)	0.161
Blastulation rate (%) (mean SD)	47.5 (33)	41.6 (34)	0.805
Euploidy rate (%) (mean, SD)	28.8 (34)	26.2 (38)	0.870
Cycle cancellation (*n*,%)	57 (15.8)	11 (10.2)	0.163
Premature ovulation (*n*, %)	1 (0.3)	3 (3.0)	**0.019**

Legend: Cycle characteristics and outcomes compared between the two CC-based protocols. Statistical analysis performed with Student’s *t*-test for stimulation days, estradiol and progesterone levels, and endometrial thickness. Linear regression controlling for patient age, race, AMH, AFC, BMI, and parity performed for number of follicles $\ge$ 14 mm on trigger day, number of total and mature oocytes retrieved, and embryologic outcomes. Blastulation and euploidy rate are shown only for cycles that were cultured to blastocyst (cleavage cycles excluded). Premature ovulation and cycle cancellation analyzed with the chi-squared test. Statistically significant *P*-values are presented in bold

Rates of cycle cancellation were not significantly different between the groups when considering cancellation for any reason, including low or no response (Table 2). Premature ovulation rates were significantly lower in the long CC group (0.3%) as compared to the 5-day CC + GnRH antagonist group (3.0%) (relative risk ratio 0.32, 95% confidence interval (0.18–0.58), *P* = 0.019).

Embryo outcomes are shown in Table 2; there were no differences observed between the two protocols. Live birth rate in the long CC group after single euploid frozen embryo transfer was 59.3%.

## Discussion

Key findings of the current study are that a long CC protocol leads to similar oocyte yield and embryologic outcomes in DOR patients as compared to a 5-day CC + GnRH antagonist protocol. As GnRH antagonist is not necessary, this protocol could reduce costs and injection burden. The long CC protocol also effectively prevented premature ovulation, as it only occurred in one patient, though this analysis was not powered to detect a difference in premature ovulation given the rarity of this event and larger studies are required.

Prior studies support that the addition of CC to high-dose recombinant gonadotropins in GnRH antagonist protocols increases total serum FSH, translating to increased oocyte yield in patients with anticipated poor response [6, 9]. Jovanovic et al. analyzed consecutive GnRH antagonist cycles in the same patient, one without the addition of CC and one with the addition of a 5-day course of CC keeping the rFSH dose constant. They found that the addition of CC increased oocyte yield and estrogen levels while decreasing cycle cancellation. [6] While our study did not measure FSH levels, it suggests that continuing CC throughout the entire cycle further increases estrogen levels and total oocytes retrieved, but leads to a similar number of mature oocytes retrieved. It is worth noting, however, that the baseline AFC in the long CC group was slightly lower than the 5-day CC + GnRH antagonist group, thus this study cannot rule out the potential clinical benefit of the long CC protocol for mature oocyte yield.

While mature oocyte yield was similar between the two CC-based protocols in this study, the use of CC to prevent ovulation and supplant the role for GnRH antagonists is an added benefit of the long CC protocol. Oral medications to prevent premature ovulation instead of GnRH antagonist agents are an attractive alternative to improve cost and injection burden as well as patient satisfaction. Progestin-primed ovarian stimulation is one such regimen that has been successfully utilized to prevent the endogenous LH surge [10–12]; however, a long CC protocol has the added benefit as compared to progestins of enhanced pituitary FSH release in poor responders. Cost savings are estimated to be on the order of several hundred dollars due to the GnRH antagonist but exact costs depend on pharmacy, insurance coverage, and medication availability.

Patients with poor response to ovarian stimulation and those with diminished ovarian reserve are particularly at risk of premature ovulation [13, 14]. There is considerable heterogeneity in studies evaluating rates of premature ovulation amongst different ovarian stimulation protocols, owing to differences in definition of premature LH rise/surge versus actual clinical evidence of ovulation as well as ovarian reserve parameters of the study population. This definition is particularly relevant when evaluating CC to prevent ovulation, as CC does increase total LH during stimulation but seems to prevent the true surge pattern necessary for ovulation [5, 15–18]. Nevertheless, the majority of studies suggest that in GnRH antagonist protocols the rate of true premature ovulation is low overall, approximately 1%; however, it may be as high as 5–6% in patients with diminished ovarian reserve [14, 19–21]. Our results are consistent with those previously reported in the literature in patients with diminished ovarian reserve but also suggest that CC may be a more effective method pending future larger scale studies.

The use of CC to simultaneously achieve ovarian stimulation and prevent premature ovulation has been utilized previously for minimal stimulation protocols [22–25]. Teramoto and Kato reported on over 40,000 minimal stimulation cycles, of which only 2–3% resulted in premature ovulation [23]. Interestingly, they also found that LH levels were more strongly suppressed with higher estradiol levels (particularly over 1000 pg/mL). This may suggest that CC may have an even more potent effect on ovulation prevention in conventional high-dose gonadotropin stimulation such as in this study as compared to minimal stimulation protocols.

One concern with a long CC protocol is the effect on the endometrium and endometrial receptivity, given estrogen receptor antagonism at the level of the endometrium. None of the patients in this study who participated in the long CC protocol underwent fresh transfer; however, we did not see a significant difference in endometrial thickness between the two groups. While this may be irrelevant for clinics who routinely practice freeze-all embryo transfer, further study to evaluate endometrial receptivity and expression patterns in patients who undergo long CC protocols would be useful.

There are several limitations of the current study. First, there was no standard definition used for diminished ovarian reserve or poor responder and rather was decided by the treating physician. Therefore, it is difficult to generalize the results of this study to specific patient populations or subgroups with poor anticipated response by age or ovarian reserve parameters. Second, this study included patients undergoing IVF with different approaches, i.e., blastocyst culture with PGT-A and frozen embryo transfer versus cleavage-stage fresh transfer. While no significant differences were detected in embryo outcomes, further prospective study is warranted with a more standardized IVF approach. Due to this, live birth was not able to be compared between the two groups, but the 59.3% live birth rate with single euploid FET in the long CC group is consistent with published live birth rates with the use of single euploid embryo transfer. Pregnancy outcomes were also not available for study. Finally, LH was not measured throughout the cycle, and premature ovulation was defined by clinical parameters. This limited our ability to fully elucidate LH patterns in the long CC protocol, and further study is necessary to fully understand this.

In conclusion, a long CC protocol may be a reasonable option for patients undergoing IVF with a freeze-all approach, as it is effective in terms of oocyte yield and embryonic development, cost-conscious, and patient-friendly by reducing injection burden.

## Data Availability

Datasets can be requested from the corresponding author.
